# Alterations of functional connectivities associated with autism spectrum disorder symptom severity: a multi-site study using multivariate pattern analysis

**DOI:** 10.1038/s41598-020-60702-2

**Published:** 2020-03-09

**Authors:** Xingdan Liu, Huifang Huang

**Affiliations:** School of Computer and Information Technology, Beijing Jiaotong University, Beijing, 100044 China

**Keywords:** Autism spectrum disorders, Functional magnetic resonance imaging, Autism spectrum disorders

## Abstract

Autism spectrum disorder (ASD) is a highly heterogeneous neurodevelopmental disorder. The estimation of ASD severity is very important in clinical practice due to providing a more elaborate diagnosis. Although several studies have revealed some resting-state functional connectivities (RSFCs) that are related to the ASD severity, they have all been based on small-sample data and local RSFCs. The aim of the present study is to adopt multivariate pattern analysis to investigate a subset of connectivities among whole-brain RSFCs that are more contributive to ASD severity estimation based on large-sample data. Regression estimation shows a Pearson correlation value of 0.5 between the estimated and observed severity, with a mean absolute error of 1.41. The results provide obvious evidence that some RSFCs undergo notable alterations with the severity of ASD. More importantly, these selected RSFCs have an abnormality in the connection modes of the inter-network and intra-network connections. In addition, these selected abnormal RSFCs are mainly associated with the sensorimotor network, the default mode network, and inter-hemispheric connectivities, while exhibiting significant left hemisphere lateralization. Overall, this study indicates that some RSFCs suffer from abnormal alterations in patients with ASD, providing additional evidence of large-scale functional network alterations in ASD.

## Introduction

Autism spectrum disorder (ASD), a prevalent, complex and highly heterogeneous neurodevelopmental disease that often occurs in infants and young children, has and imposes abnormal effects on children’s social interactions, communication, and many other behavioural and cognitive functions^1^. It is reported that one in every 68 American children is affected by some form of ASD, and the number of children with ASD has increased by 78% compared with the previous decade^2^. ASD brings a heavy psychological and economic burden to the patients, their families, and society^3^. Therefore, accurate, early diagnosis of ASD and appropriate interventions may greatly improve the present status.

The current diagnosis of ASD is primarily symptom-based. Specifically, the physician makes a comprehensive assessment of the patient according to the Autism Diagnostic Observation Schedule (ADOS)^4,5^ and the Autism Diagnostic Interview-Revised (ADI-R)^6,7^. He or she determines whether the subject has ASD based on the assessment scores; however, this diagnostic procedure lacks neurobiological biomarkers. Resting-state functional magnetic resonance imaging (rs-fMRI) has become a powerful tool for exploring intrinsic functional connectivity in the human brain. Many studies have been committed to finding functional biomarkers to accurately distinguish between ASD patients and typically developing (TD) individuals through rs-fMRI^8–11^ as a form of qualitative diagnosis of ASD.

However, a quantitative estimation of the ASD severity may be more crucial than the qualitative diagnosis in clinical practice. Quantitative estimation requires establishing a relationship between functional or structural abnormalities and disorder severity measures. The severity of a disorder is a continuous, non-categorical measure upon which a more elaborate diagnosis can be made. ASD severity estimation has important advantages. First, there are different treatment options according to different ASD severities in clinical practice, and a precise ASD severity assessment can clinically guide the intervention treatment and prognosis. Second, a precise assessment of ASD severity can help us to understand the variation in biomarkers with respect to different degrees of severity, revealing the basis of the associated neurophysiological changes. Third, estimating ASD severity can provide more comprehensive and personalized information for ASD individuals. Finally, the measurement of the severity of the disease could further facilitate the search for neurobiological markers to aid in the qualitative diagnosis of ASD. Specifically, the severity may be a considerable source of heterogeneity that affects the accuracy of ASD qualitative diagnosis^12^. It is not surprising that not only ASD patients but also TD individuals exhibit some autism-like symptoms in many behaviours, which is likely to obscure the neurological abnormalities associated with ASD symptoms and prevent the accurate identification of ASD individuals using MRI data to a great extent.

To the best of our knowledge, only a small number of studies have carried out ASD severity estimation using MRI data. Based on structural MRI (sMRI) data, several studies have estimated the ASD severity and revealed some important brain structures^12,13^. Although many previous studies have reported correlations between abnormalities in brain function and the severity of ASD with rs-fMRI data, only several studies have used local resting-state functional connectivities (RSFCs) for individual ASD severity estimation based on small-sample data^14,15^. Uddin *et al*.^14^ only used voxel information in the salience network to estimate the severity in 20 ASD patients, and Yahata *et al*.^15^ used 16 RSFCs determined in ASD diagnosis to estimate the severity in 58 ASD individuals. Few studies have estimated continuous ASD severity using whole-brain RSFCs based on large-sample data.

The purpose of this study is to investigate the whole-brain RSFCs that contribute the most to ASD severity estimation based on large-sample data from multiple sites. We conducted the study using multivariate pattern analysis on 174 ASD rs-fMRI data extracted from three sites. Specifically, we evaluated the RSFCs between 116 regions of interest (ROIs) with Pearson correlation coefficients and then performed ASD severity estimation based on the surviving RSFCs using linear support vector regression (SVR). In the current study, the 116 ROIs in the Automated Anatomical Labeling (AAL) template were divided into six common functional networks according to the BrainNet Viewer software^16^ (see Supplementary Table 1 for details): the default mode network (DMN), the execution and attention network (EAN), the sensorimotor network (SMN), the visual network (Visual), the subcortical nuclei (SBN) regions and the cerebellum (Cerebel). To obtain the extent and pattern of the alterations in the functional connectivities associated with ASD severity, we analysed the RSFCs that were repeatedly selected as features in leave-one-out cross-validation (LOOCV) for ASD severity estimation. More importantly, the contributions of the RSFCs were determined to evaluate their importance. Finally, we summarized the contributions of different divisions of RSFCs to ASD severity and discussed these results in detail.

## Results

### Higher correlation value between the estimated and observed severity

**Figure 1:**
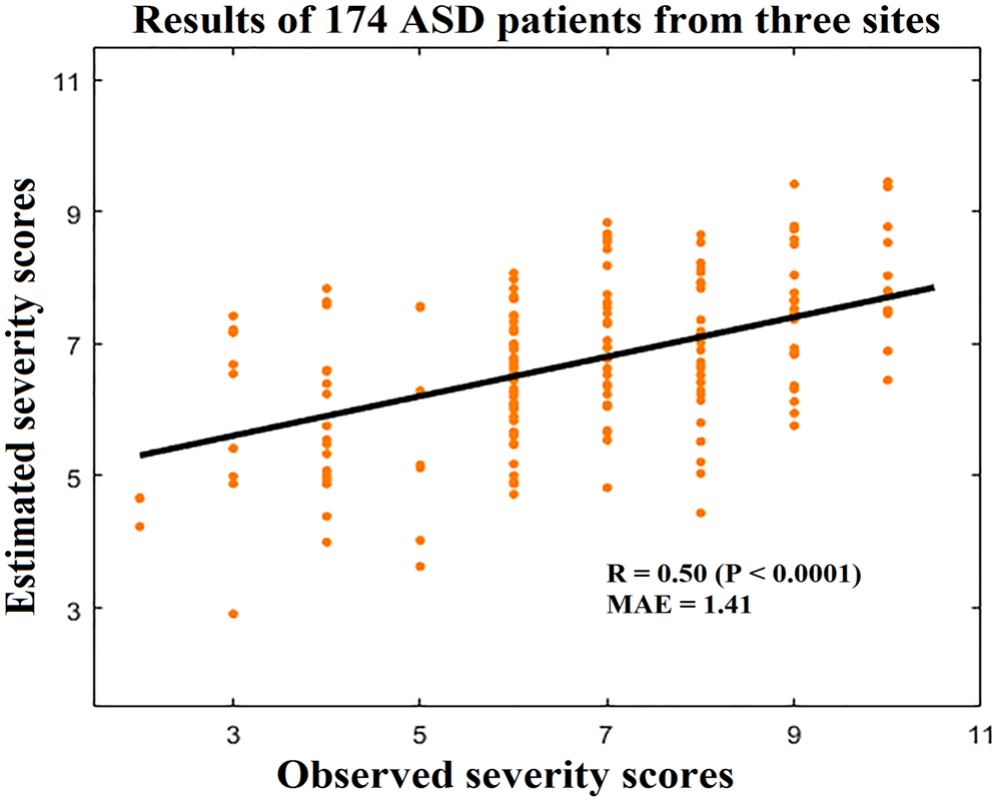
Correlation between the estimated and observed severity scores of 174 ASD patients from three sites. R: Pearson correlation coefficient; P: significance; MAE: mean absolute error.

Using the linear SVR method, the regression results of 174 ASD patients from three sites demonstrate that some RSFCs contain important information about severity. Specifically, the Pearson correlation value (R) between the estimated and observed severity scores is 0.50 (P < 0.0001), and the mean absolute error (MAE) is 1.41 (Fig. 1).

### Analysis of contributions of selected RSFCs from different perspectives

**Figure 2:**
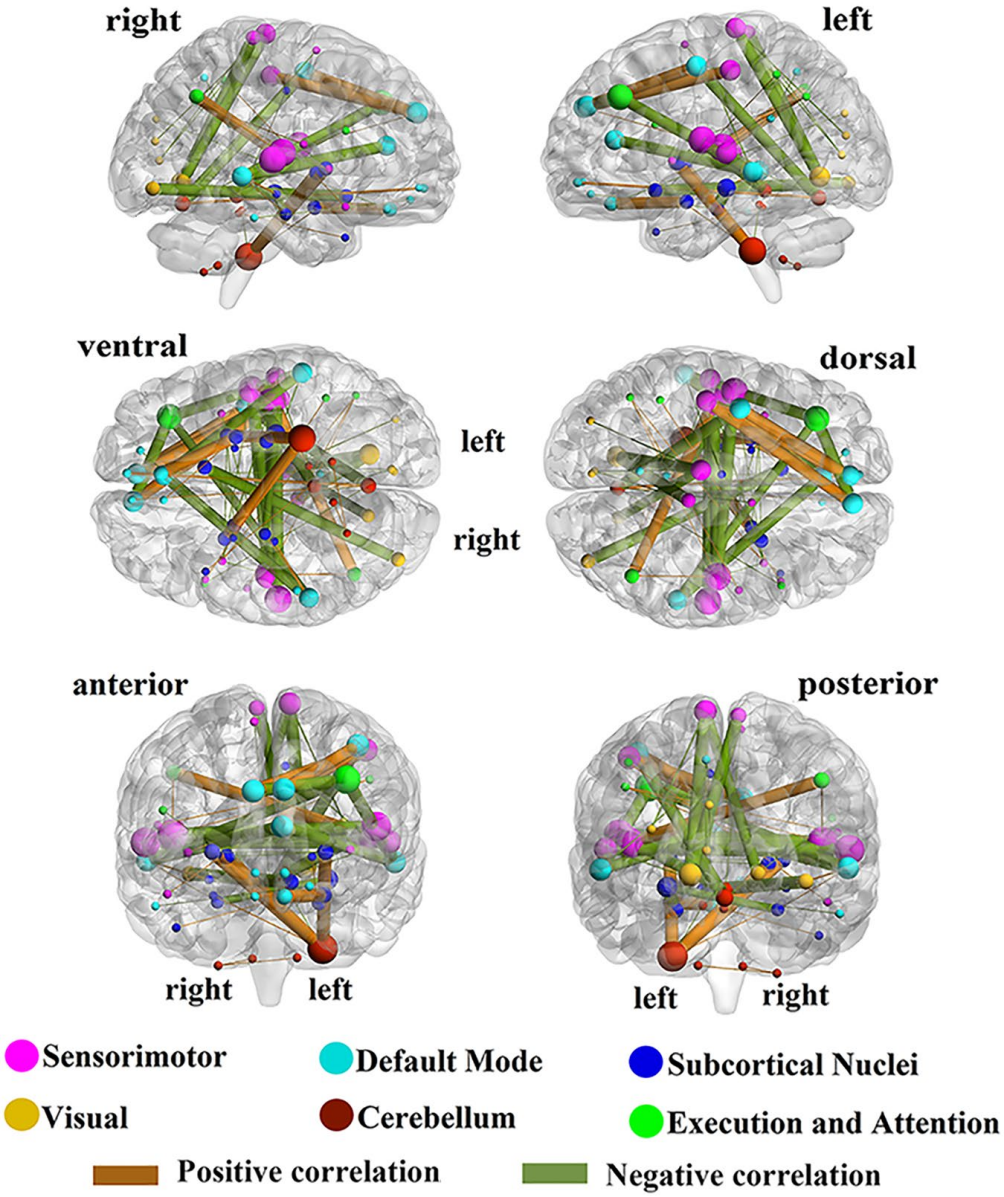
Contributions and distribution of selected RSFCs in the whole brain. The selected RSFCs are displayed on a surface rendering of the brain using the BrainNet Viewer software. The orange/green lines indicate that the RSFCs were positively/negatively correlated with the severity scores. The 116 ROIs were divided into six different functional networks. The contributions of the selected RSFCs/ROIs are reflected by their thicknesses/sizes. Specifically, the contribution of an RSFC is defined by the ratio of the folds from the LOOCV in which the RSFC was chosen as a feature to all folds, and the contribution of the ROI is evaluated by the contributions of all the RSFCs associated with it.

**Figure 3:**
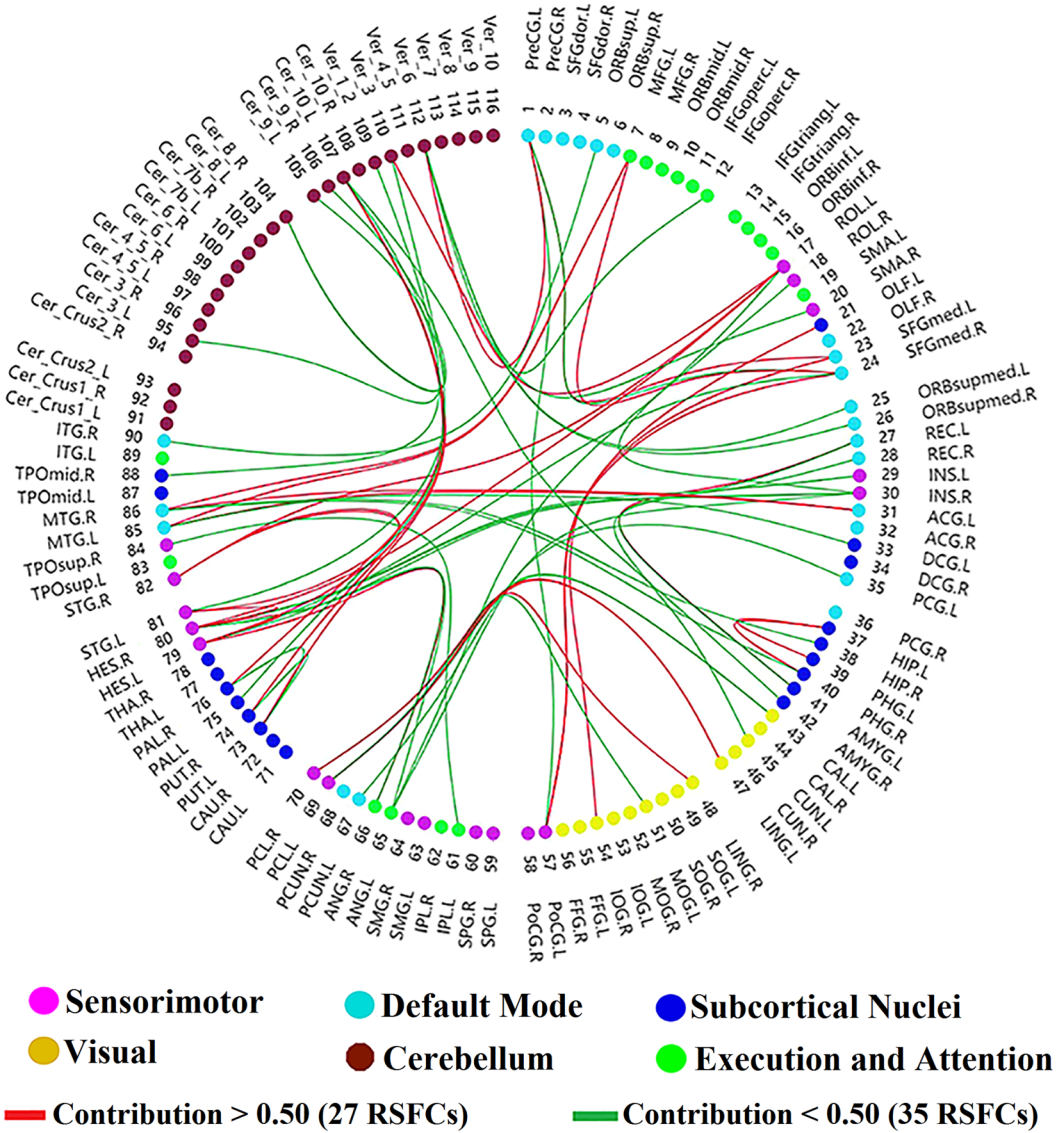
Distribution of all selected RSFCs based on the AAL brain template. Sixty-two RSFCs out of the 6670 RSFCs were selected at least once, and 59 ROIs were related to the selected RSFCs. The contribution of an RSFC is defined by the ratio of the folds from the LOOCV in which the RSFC was chosen as a feature to all folds. Red/green lines indicate the contributions of RSFCs above/below 0.5. All 116 ROIs were assigned to six different functional networks represented by the six different colours.

**Figure 4:**
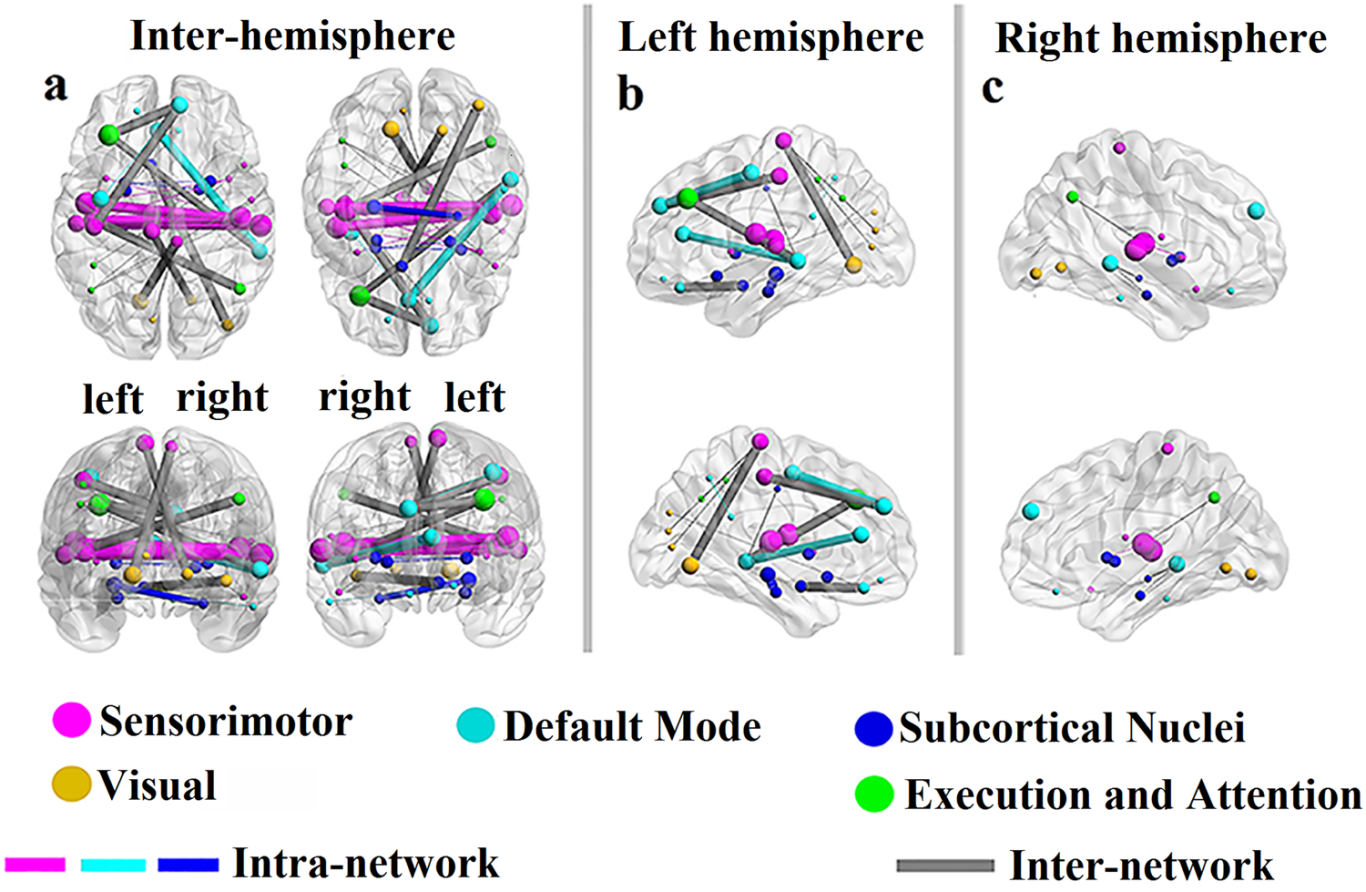
Distribution of intra-network and inter-network RSFCs in the cerebral cortex from three perspectives. (**a**) only shows the inter-hemispherical RSFCs. (**b**),(**c**) draw the RSFCs within the left and right hemisphere, respectively. An RSFC whose two ends come from the same network is defined as an intra-network connection, and it has the same colour as the associated ROIs. An RSFC whose two ends come from different networks represents an inter-network connection and is coloured grey. Here, the intra-network RSFCs only involve three functional networks: the SMN, DMN and SBN. The inter-network RSFCs are widely distributed among five functional networks in the cerebral cortex.

The contributions of the RSFCs to ASD severity estimation can be analysed. Since the experiment was performed using LOOCV and the training set changes for each fold, the selected RSFCs based on the training set are diverse from fold to fold. Sixty-two RSFCs out of the 6670 RSFCs were selected at least once in all folds (detailed information is listed in Supplementary Table S2), and 59 ROIs were related to the selected RSFCs, as displayed in Fig. 2. We define the contribution of an RSFC by the ratio of the folds in which the RSFC is chosen as a feature to the total number of folds^17^. In Fig. 2, the orange/green lines indicate positive/negative correlations between the RSFCs and observed scores, respectively. The thicknesses of the RSFC lines and the sizes of the nodes are in proportion to their contributions. In Fig. 3, the red/green lines indicate contributions of RSFC above/below 0.5, and Table 1 gives the details of the 27 RSFCs whose contributions are above 0.5. Figure 4 shows the distribution of the contributions of intra-network and inter-network RSFCs in the cerebral cortex from three perspectives: the inter-hemisphere, the left hemisphere (LH), and the right hemisphere (RH) contributions.

**Table 1:** Details of the RSFCs whose contributions are above 0.5 (27 RSFCs).

ROI1	Coordinates	BL	Net	ROI2	Coordinates	BL	Net	R	Contribution
Name (BA)				Name (BA)					
**Positive RSFCs**									
Cerebelum_10_L(107)	−22, −33, −41	CRBL10.L	Cerebel	PUT.R(74)	27, 4, 2	Insula	SBN	0.33	1.00
Cerebelum_10_L(107)	−22, −33, −41	CRBL10.L	Cerebel	PUT.L(73)	−23, 3, 2	Insula	SBN	0.3	1.00
SFGmed.R(24)	9, 50, 30	prefrontal	DMN	PoCG.L(57)	−42, −22, 48	Parietal	SMN	0.30^a,b^	1.00^c^
Cerebelum_10_L(107)	−22, −33, −41	CRBL10.L	Cerebel	PAL.R(76)	21, 0, 0	Insula	SBN	0.29	1.00
HES.L(79)	−41, −18, 9	Temporal	SMN	ANG.R(66)	45, −59, 38	Parietal	EAN	0.27^a^	1.00^c^
SFGmed.L(23)	−4, 49, 30	prefrontal	DMN	PoCG.L(57)	−42, −22, 48	Parietal	SMN	0.26^a,b^	1.00^d^
REC.L(27)	−5, 37, −18	prefrontal	DMN	AMYG.L(41)	−23, 0, −17	Temporal	SBN	0.25^b^	1.00^d^
SFGmed.L(23)	−4, 49, 30	prefrontal	DMN	PreCG.L(1)	−38, −5, 50	Frontal	DMN^*^	0.24^b^	0.98^d^
**Negative RSFCs**									
HES.L(79)	−41, −18, 9	Temporal	SMN	HES.R(80)	45, −17, 10	Temporal	SMN^*^	−0.32^a^	1.00^c^
HES.L(79)	−41, −18, 9	Temporal	SMN	STG.R(82)	58, −21, 6	Temporal	SMN^*^	−0.29^a^	1.00^c^
STG.R(82)	58, −21, 6	Temporal	SMN	STG.L(81)	−53, −20, 7	Temporal	SMN^*^	−0.28^a^	1.00^c^
STG.R(82)	58, −21, 6	Temporal	SMN	ROL.L(17)	−47, −8, 13	Frontal	SMN^*^	−0.28^a^	1.00^c^
HES.R(80)	45, −17, 10	Temporal	SMN	ROL.L(17)	−47, −8, 13	Frontal	SMN^*^	−0.27^a^	1.00^c^
STG.L(81)	−53, −20, 7	Temporal	SMN	HES.R(80)	45, −17, 10	Temporal	SMN^*^	−0.27^a^	1.00^c^
PreCG.L(1)	−38, −5, 50	Frontal	DMN	Vermis_6(112)	1, −67, −15	Vermis6	Cerebel	−0.25^b^	1.00
OLF.L(21)	−8, 15, −11	prefrontal	SBN	IOG.R(54)	38, −81, −7	Occipital	Visual	−0.25	1.00^c^
HIP.L(37)	−25, −20, −10	Temporal	SBN	PHG.L(39)	−21, −15, −20	Temporal	SBN^*^	−0.26	0.99^d^
MTG.R(86)	57, −37, −1	Temporal	DMN	ACG.L(31)	−4, 35, 13	Insula	DMN^*^	−0.25^b^	0.99^c^
LING.L(47)	−14, −67, −4	Occipital	Visual	PCL.L(69)	−7, −25, 70	Parietal	SMN	−0.25^a^	0.99^d^
LING.L(47)	−14, −67, −4	Occipital	Visual	PCL.R(70)	7, −31, 68	Parietal	SMN	−0.25^a^	0.99^c^
SFGmed.R(24)	9, 50, 30	prefrontal	DMN	MFG.L(7)	−33, 32, 35	prefrontal	EAN	−0.24^b^	0.99^c^
MTG.L(85)	−55, −33, −2	Temporal	DMN	MFG.L(7)	−33, 32, 35	prefrontal	EAN	−0.24^b^	0.98^d^
ROL.L(17)	−47, −8, 13	Frontal	SMN	Vermis_3(110)	1, −39, −11	Vermis3	Cerebel	−0.24^a^	0.93
MTG.L(85)	−55, −33, −2	Temporal	DMN	ACG.L(31)	−4, 35, 13	Insula	DMN^*^	−0.23^b^	0.87^d^
LING.R(48)	16, −66, −3	Occipital	Visual	PCL.L(69)	−7, −25, 70	Parietal	SMN	−0.24^a^	0.85^c^
MTG.R(86)	57, −37, −1	Temporal	DMN	MFG.L(7)	−33, 32, 35	prefrontal	EAN	−0.23^b^	0.77^c^
HIP.L(37)	−25, −20, −10	Temporal	SBN	PHG.R(40)	25, −15, −, −20	Temporal	SBN^*^	−0.23	0.64^c^

Abbreviations: “BA” indicates the AAL brain area; “.L”, left-hemisphere; “.R”, right-hemisphere; “BL”: brain lobe. “Coordinates” refer to the AAL coordinate. A positive/negative “R” value indicates a positive/negative correlation between the RSFC and the observation score, and |R| > 0.17 corresponds to P < 0.05 (uncorrected). The “Contribution” of the RSFC is calculated as the ratio of the LOOCV folds in which the RSFC was chosen as a feature to all folds. “*” indicates the intra-network RSFCs, and the remaining RSFCs are inter-network. “^a^” indicates SMN-related RSFCs. “^b^” indicates DMN-related RSFCs. “^c^” indicates inter-hemispheric RSFCs (except cerebellum), and “^d^” indicates RSFCs within the left hemisphere. DMN, default mode network; SMN, sensorimotor network; SBN, subcortical nuclei regions; EAN, execution and attention network; Visual, visual network; Cerebel, cerebellum.

### Contributions of different divisions of RSFCs to ASD severity

**Figure 5:**
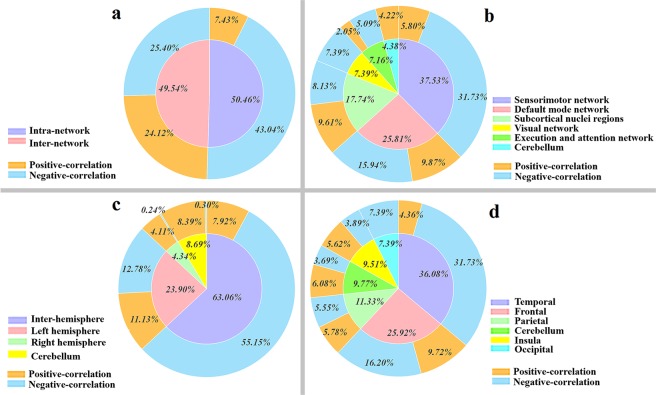
Quantitative summary of the contributions of different divisions of RSFCs to ASD severity. Each pie slice in the inner ring indicates a division, and the contribution of each division is the normalized sum of the contributions of all the connectivities whose ROIs are within the division: (**a**) reflects the contributions of the inter-network and intra-network connectivities. (**b**) indicates the contributions of connectivities related to the six functional networks. (**c**) indicates the contributions of the inter-hemispheric, left hemisphere, right hemisphere and the cerebellar connectivities, respectively. (**d**) shows the contributions of connectivities related to the six brain lobes. The outer pie slices indicate the contributions of the positive/negative-correlation RSFCs corresponding to the pie slice in the inner ring.

Figure 5 summarizes the contributions of RSFCs from four different divisions to ASD severity. Specifically, each pie slice in the inner ring indicates a division, and the contribution of each division is the normalized sum of the contributions of all the connectivities whose ROIs are located in that division. Furthermore, RSFCs that are positively correlated with the severity scores are considered “positive-correlation connectivities”, while RSFCs that are negatively correlated with the severity scores are considered “negative-correlation connectivities”. The outer pie slices display the contributions of the positive/negative-correlation connectivities corresponding to the pie slice in the inner ring.

### Important characteristics of the selected RSFCs regarding ASD severity

Summarizing the above results, the major characteristics of the 62 selected RSFCs (selected at least once in the LOOCV) are as follows: (1) A majority of the selected RSFCs are negatively correlated with ASD severity, accounting for nearly 70% of the total contribution (Fig. 2, the outer ring of Fig. 5, and Table 1). (2) The intra-network and inter-network connectivities have an equal contribution to ASD severity (50.46% and 49.54%, Fig. 5a). However, the positive- and negative-correlation connectivities contribute almost equally to inter-network RSFCs (24.12% and 25.40%, Fig. 5a). The contribution to intra-network RSFCs is mainly from negative-correlation connectivities (43.04% of 50.46%, Fig. 5a). (3) Among the six functional networks, the RSFCs associated with the SMN and DMN contribute more than 60% (37.53% and 25.81%, Fig. 5b), and these RSFCs are mainly negatively correlated with the severity. (4) Inter-hemispherical RSFCs have a significant contribution to the severity, and most of them are also negative-correlation connectivities (55.15% of 63.06%, Fig. 5c). (5) The number and contributions of the RSFCs within the LH are much greater than those of the RSFCs in the RH, showing an obvious LH lateralization (Figs. 4b, c, and 5c). (6) The RSFCs associated with the temporal and frontal lobes have important contributions (36.08% and 25.92%, Fig. 5d).

### Important ROIs associated with selected RSFCs

Table 2 lists the details of the top 20 ROIs according to their contributions to the severity estimation (the selection frequency is 0.33). It is obvious that the selection frequency is quite high, meaning that the robustness of the features is reasonable. The ROIs related to language systems, such as the Heschl’s gyri (HES_L(79), HES_R(80)), the superior temporal gyri (STG.L(81), STG.R(82)), the middle temporal gyri (MTG.L(85), MTG.R(86)), the left Rolandic operculum (ROL.L(17)), and the medial superior frontal gyri (SFGmed.R (24), SFGmed.L (23)), contribute greatly to the severity estimation.

**Table 2:** The top 20 ROIs for ASD severity estimation.

ROI Name	Coordinates	BL	Network	Contribution
HES.L(79)	−41, −18, 9	Temporal	SMN	1.00^a,c^
HES.R(80)	45, −17, 10	Temporal	SMN	1.00^a,c^
Cerebelum_10_L(107)	−22, −33, −41	CRBL10.L	Cerebel	0.99
STG.R(82)	58, −21, 6	Temporal	SMN	0.98^a,c^
ROL.L(17)	−47, −8, 13	Frontal	SMN	0.96^b,c^
MFG.L(7)	−33, 32, 35	Prefrontal	EAN	0.89^b^
STG.L(81)	−53, −20, 7	Temporal	SMN	0.66^a,c^
PoCG.L(57)	−42, −22, 48	Parietal	SMN	0.65^c^
SFGmed.R(24)	9, 50, 30	Prefrontal	DMN	0.65^b,d^
PreCG.L(1)	−38, −5, 50	Frontal	DMN	0.65^b,d^
LING.L(47)	−14, −67, −4	Occipital	Visual	0.65
SFGmed.L(23)	−4, 49, 30	Prefrontal	DMN	0.64^b,d^
PCL.L(69)	−7, −25, 70	Parietal	SMN	0.63^c^
MTG.L(85)	−55, −33, −2	Temporal	DMN	0.62^a,d^
ACG.L(31)	−4, 35, 13	Insula	DMN	0.61^d^
MTG.R(86)	57, −37, −1	Temporal	DMN	0.61^a,d^
HIP.L(37)	−25, −20, −10	Temporal	SBN	0.53 ^a^
Vermis_6(112)	1, −67, −15	Vermis6	Cerebel	0.39
ANG.R(66)	45, −59, 38	Parietal	EAN	0.34
PUT.L(73)	−23, 3, 2	Insula	SBN	0.33

Abbreviations: “.L”, left-hemisphere; “.R”, right-hemisphere; “BL”: brain lobe. “Coordinates” refer to the AAL coordinates. The contribution of the ROI is normalized to the 0–1 range through min-max normalization. “^a^” indicates ROIs within the temporal lobe. “^b^” indicates ROIs within the frontal lobe (including the prefrontal cortex). “^c^” indicates ROIs within the SMN. “^d^” indicates ROIs within the DMN. DMN, default mode network; SMN, sensorimotor network; SBN, subcortical nuclei regions; EAN, execution and attention network; Visual, visual network; Cerebel, cerebellum.

## Discussion

To the best of our knowledge, only a small number of studies have estimated ASD severity based on fMRI data^14,15^. Uddin *et al*.^14^ adopted a multivariate sparse regression method to estimate the disease severity in 20 ASD individuals with a correlation value of 0.36, and they found that the salience network was associated with ASD severity. Yahata *et al*.^15^ estimated the disease severity in 58 ASD patients with a correlation value of 0.44 based on 16 functional connectivity measurements determined in the ASD diagnosis. However, studies using fMRI data to estimate the ASD severity are based on small-sample data, and there has been no large-sample study to date on ASD severity estimation using multivariate pattern analysis through whole-brain RSFCs. In the current study, we used multivariate pattern analysis to investigate which connectivities among whole-brain RSFCs contribute more to ASD severity based on large-sample data from three sites. The results demonstrate that the ASD severity could be estimated with comparatively high accuracy: based on 174 ASD patients from three imaging sites, the Pearson correlation value between the estimated and observed scores was R = 0.50 (P < 0.0001), and the MAE was 1.41 (Fig. 1). Our results provide strong evidence that some RSFCs undergo significant alterations according to the difference in the degree of ASD severity and contain important information for ASD severity estimation.

To validate the robustness of our results, a 10-fold CV was repeated ten times to estimate the severity of ASD (the mean results are listed in Supplementary Table S3). With the selected threshold (P = 0.0023), the MAE (1.57) of the 10-fold CV does not increase substantially compared to the MAE (1.41) of the LOOCV. However, the value of the Pearson correlation coefficient decreases from 0.50 (LOOCV) to 0.36 (10-fold CV). This means that to some extent, the LOOCV method may produce overfitting because the training data are almost the same each time. On the other hand, the current sample size may not be sufficient to overcome the unstableness of the 10-fold CV. Nevertheless, the selected RSFCs still show strong robustness. Specifically, for the selected top RSFCs, both LOOCV and 10-fold CV generate consistent results. As shown in Supplementary Table S4, all 62 RSFCs selected with the LOOCV are also selected in the 10-fold CV (120 RSFCs). The top 27 RSFCs (contribution > 0.50) in the LOOCV are also the top 27 RSFCs (contribution > 0.36) in the 10-fold CV, and the contributions of the top 22 RSFCs in the 10-fold CV are above 0.50. The results demonstrate that the selected RSFCs in the LOOCV are still robust in the 10-fold CV.

Some intra-network and inter-network connectivities are abnormal in ASD, and these abnormal connectivities are important for ASD severity estimation. Specifically, Dosenbach *et al*.^18^ reported that the maturity trajectories of the normal brain are characterized mainly by negative-correlation inter-network connectivities, followed by positive-correlation intra-network connectivities. This development pattern is necessary and important for efficient information processing^18^. Some studies have reported abnormal intra-network and inter-network connectivities in ASD^19,20^. For example, Morgan *et al*.^19^ suggested that ASD evidently combined increased inter-network connectivity and reduced intra-network connectivity in children and adults, and this abnormal connection mode may cause social-cognitive deficits and is also related to ASD symptom severity. In the current study, the inter-network and intra-network connectivities revealed two major points. First, the inter-network and intra-network connectivities tended to have an approximately equal contribution (50.46% and 49.54%, Fig. 5a), which is different from the maturity trajectories of the normal brain, wherein inter-network connectivity plays a more important role than intra-network connectivity, as reported by Dosenbach *et al*.^18^. Second, the positive- and negative-correlation RSFCs showed nearly the same contributions to the inter-network connectivity (24.12% and 25.4%, Fig. 5a). The contribution to intra-network connectivity is mainly from negative-correlation RSFCs (43.03% of 50.46%, Fig. 5a). Overall, this abnormal inter-network and intra-network connectivity pattern reveals three important pieces of information: (1) It is different from the normal brain maturity trajectories reported by Dosenbach *et al*.^18^, suggesting that the brain development of ASD patients deviates from normal developmental trajectories as early as childhood. This atypical development could influence normal information processing, which may cause different levels of ASD symptoms. (2) It is similar to the results of Morgan *et al*.^19^ They reported an abnormal pattern of intra-network and inter-network connectivity related to ASD symptom severity, which supports our experimental results. (3) It provides direct evidence that abnormal inter-network and intra-network connectivities could influence ASD severity and provide a wealth of information about ASD.

In the current study, many SMN- and DMN-related RSFCs had significant contributions (37.53% and 25.81, Fig. 5b) to the ASD severity estimation compared to those related to other networks, and most of these RSFCs were negative-correlation connectivities. We believe that this phenomenon might cause damage to the normal development of network-related function and may contain important information about ASD dysfunctions. In the next two paragraphs, we will discuss the important impact of the SMN and DMN on estimating ASD severity.

The SMN is a large-scale brain network that is activated during motor tasks and plays an important role in ASD-related studies. A series of SMN-related deficits, such as social communication and interaction difficulties, atypical sensory responsivity, and repetitive and restricted behaviours, have been incorporated into the present diagnostic criteria of ASD^21^. Although most ASD patients show abnormal development of the motor system, studies about motor impairments in ASD have received far less attention than those about core social communication and cognitive damage. However, Mosconi *et al*.^22^ emphasized the importance of research on sensorimotor dysfunctions in ASD, and they suggested that sensorimotor deficits occur before social and communication deficits and are primary features of ASD. Some studies have also noted that early signs of ASD-related damage might first appear in the motor system, behaving as motor delays^22–24^, and that the deficits in sensorimotor processing in ASD could further influence the development of more advanced functions^25–27^. For example, Hannant *et al*.^25^ mentioned that sensorimotor deficits associated with core ASD symptoms have a cascading effect on advanced functions, such as social, communicative and emotional development. In addition, some studies have demonstrated that sensorimotor skills are associated with the severity of ASD symptoms^21,28^. Specifically, Tavassoli *et al*.^28^ indicated that reduced sensory perception is associated with a greater number of autism symptoms, which supports our experimental results: most SMN-related RSFCs were negatively correlated with severity scores (Fig. 5b, Table 1). The problem of sensorimotor deficits in ASD should receive more attention in future studies.

As a baseline state in brain function^29^ and a sensitive biomarker of mental disease^30^, the DMN has been widely reported to show abnormalities in psychiatric disorders, and the abnormalities of DMN are also related to symptom severity^31^. Importantly, abnormalities of the DMN are regarded as prominent ASD neurobiological features^32^. Specifically, it is well known that ASD is characterized by impairments in social communication and interaction, and the DMN plays a vital role in socially relevant stimuli because of its involvement in the mentation of self-reflective thought and in the consideration of the perspective of others^32^. Some studies have reported that the widely decreased RSFCs of the DMN in ASD not only contribute to the core deficits of ASD but also have a great influence on symptom severity^33–35^. For example, Assaf *et al*.^34^ indicated that the core areas and subnetworks of the DMN in ASD showed decreased functional connectivity and that their functional connectivity magnitudes were negatively correlated with the severity of social and communication deficits in ASD. In the current study, the results also show that most DMN-related RSFCs were negatively correlated with the severity of ASD.

Many studies have revealed the widespread decline of inter-hemispheric connectivity in ASD, and these abnormal RSFCs not only affect advanced functions but also have a significant effect on the severity of the disease^36–38^. Specifically, Kikuchi *et al*.^38^ indicated a significant reduction in the connectivities between the left-anterior and right-posterior regions in young children with ASD, and these connectivities were significantly negatively correlated with clinical severity. Dinstein *et al*.^37^ also showed that toddlers with ASD had weakened inter-hemispheric connectivities (that is, weak inter-hemispheric synchronization), and the strength of synchronization was negatively related to ASD severity. More importantly, Ilan *et al*. emphasized that a weakened inter-hemispheric synchronization was a notable characteristic of ASD neurophysiology and that the most obvious difference in hemispherical synchronicity was located in the language regions, especially the superior temporal gyrus (STG) and the inferior frontal gyrus (IFG). Strangely, they noted that the intensity of IFG synchronization was negatively correlated with ASD severity, but they did not find a relationship between the STG (an important language area that contains Wernicke’s area) and ASD severity. We speculate that this may be associated with the flaws in the evaluation indicators (ADOS communication scores) they used. Here, using the standardized proxy calibration severity scores, our results confirm that there is also a significant negative correlation between the STG and severity. In addition, the negative-correlation inter-hemisphere RSFCs (especially between the left and right temporal lobes) contribute more to ASD severity, far exceeding those of the single hemispheres (Figs. 4 and 5c, Table 1). In conclusion, the negative-correlation inter-hemisphere RSFCs (especially the language system connectivity) have a significant impact on ASD severity.

As shown in Figs. 4b and 4c, the LH has a clear advantage over the RH in terms of contributions to ASD severity. As ASD has widespread language and communication barriers^39,40^ and since the LH dominates the processing of language/semantic information, the LH plays a more important role in the estimation of ASD severity compared with the RH. More importantly, using inter-regional thickness features from the sMRI data, Sato *et al*.^13^ reported that the LH is more relevant for ASD severity estimation than the RH. Here, our results demonstrate that the LH is also more closely related to ASD severity than the RH based on fMRI data. Specifically, the number and contributions of the RSFCs within the LH are significantly higher than those of the RSFCs within the RH (Figs. 4b,c and 5c), which shows obvious LH lateralization in the estimation of ASD severity.

Temporal-related RSFCs (including the temporo-temporal RSFCs and the RSFCs connecting the temporal lobe to other regions) and frontal-related RSFCs (including the fronto-frontal RSFCs and the RSFCs connecting the frontal lobe to other regions) have a significant impact on ASD. In detail, the temporal lobe and frontal lobe are associated with advanced cognitive, social, and communication functions^41^, whose functional abnormalities can cause the core symptoms of ASD^42,43^. Some studies have reported reduced temporal- and frontal-related connectivity in ASD^44,45^. Specifically, Geschwind and Levitt^44^ indicated that frontal-related RSFCs are partially disconnected in ASD, and Sahyoun *et al*.^45^ found that the RSFCs related to linguistic regions between the frontal lobe and temporal lobe are weakened in high functioning autism children. In the current study, the temporal-related RSFCs and frontal-related RSFCs had an important contribution to ASD severity estimation (Fig. 5d, Table 1), and most of them were negatively associated with ASD severity.

The ROIs related to the language system have an important contribution to ASD severity estimation, especially the Heschl’s gyri (HES_L(79), HES_R(80)), the superior temporal gyri (STG.L(81), STG.R(82)), the middle temporal gyri (MTG.L(85), MTG.R(86)) and the left Rolandic operculum (ROL.L(17)), listed in Table 2. Specifically, the HES, as the most contributive ROI in both hemispheres, contains the primary auditory cortex. The STG has an important influence on auditory processing, especially the Wernicke’s area located in the back of the STG.L(81), which is an important language centre that plays an important role in understanding written and spoken language. The MTG is also considered to be language-dependent, especially in terms of lexical and conceptual semantics. The ROL.L not only participates in the generation of rhythm but also attends cognitive processing and physiological awakening in response to the stimulation of emotional music, and the ROL has an important impact on the social and emotional cognition in ASD^46^. In addition, the medial superior frontal gyrus (SFGmed.R (24), SFGmed.L (23)) is reported to be related to the ASD severity^47^. The precentral gyrus (PreCG.L(1)) is a key component of the motion control network and is associated with ASD severity^27^. Furthermore, it must be emphasized that the ROIs we selected using fMRI data are highly coincident with the ROIs selected using sMRI data for ASD estimation^12,13,48^. Specifically, Hazlett *et al*.^48^ estimated whether the infants were at high risk for ASD and selected the top 40 ROIs based on sMRI data using non-linear deep learning classification or linear sparse learning classification. The 40 ROIs have a considerable overlap with the ROIs we selected. More importantly, Hazlett *et al*.^48^ noted that the excessive growth of these abnormal ROIs was related to the severity of social deficits in ASD. In addition, Sato *et al*.^13^ and Moradi *et al*.^12^ used inter-regional thickness correlations and cortical thickness measurements, respectively, from sMRI to estimate ASD severity, and the important ROIs they selected also highly overlap with ours (see Supplementary Fig. S1). In conclusion, the selected ROIs have an important impact on ASD severity and further confirm the reliability of the RSFCs selected in our experiment.

## Conclusion

The aim of this study is to adopt multivariate pattern analysis to investigate which connectivities among whole-brain RSFCs contribute more to ASD severity based on large-sample data (174 ASD patients from three ABIDE I sites). By analysing the RSFCs that were repeatedly selected as features in LOOCV for estimating ASD severity, we obtained the extent and pattern of the alterations in functional connectivities associated with ASD severity. The experimental results provide strong evidence that some RSFCs associated with ASD severity truly undergo notable alterations. (1) The 62 RSFCs selected in our experiments are mainly characterized by abnormal negative-correlation RSFCs of intra-network and abnormal positive-correlation RSFCs of inter-network. This pattern may influence efficient information processing, which could cause more serious ASD severity. (2) Abnormal negative-correlation RSFCs related to the SMN play a vital role in ASD severity estimation. A similar observation is made for the DMN. (3) Inter-hemispheric RSFCs related to advanced functions significantly contribute to ASD severity, and left hemisphere lateralization reflects the dominant position of the language system in the estimation of ASD severity. (4) Temporal-related RSFCs (including the temporo-temporal RSFCs and the RSFCs connecting the temporal lobe to other regions) and frontal-related RSFCs (including the fronto-frontal RSFCs and the RSFCs connecting the frontal lobe to other regions) could influence advanced cognitive, social, and communication functions and have a significant contribution to the estimation of ASD severity. (5) More importantly, most of the selected RSFCs in our experiments are negatively correlated with severity. In conclusion, all these alterations might influence normal information processing and cause different levels of ASD symptoms. We also found corresponding evidence to support the reliability and important physiological significance of the selected RSFCs. This study not only fills in research gaps in the use of whole-brain RSFCs to estimate the severity of ASD from large-sample data, locating meaningful RSFCs for ASD severity but also indicates that these RSFCs suffer from abnormal alterations in patients with ASD, providing additional evidence of large-scale functional network alterations in ASD.

## Methods

### Dataset

The data used in this study came from the publicly available dataset, the Autism Brain Imaging Data Exchange I (ABIDE I)^49^ (http://fcon_1000.projects.nitrc.org/indi/abide/), which includes 17 independent sites. In the current study, we used 174 patient data from three sites: New York University Langone Medical Center (NYU), University of California, Los Angeles, Sample 1 (UCLA_1), and University of Utah School of Medicine (USM). According to the data repository, the initial data collection, sharing and experimental protocols of each site were approved by their local Institutional Review Board (IRB) or ethics committee, and all procedures followed the corresponding institutional regulations (i.e., IRB and regulations of NYU, UCLA, and USM). At the same time, all data processing processes followed the relevant U.S. Health Insurance Portability and Accountability Act (HIPAA) guidelines and the 1000 Functional Connectomes Project / INDI protocols. All methods were carried out in accordance with the relevant approved guidelines and regulations: all 18 HIPAA protected information were removed; all data were fully anonymized; and everyone signed informed consent, including the legal guardian of a child under the age of 18.

The subjects and imaging sites selected in the current study satisfied the following three criteria: (1) all fMRI data were preprocessed successfully; (2) the phenotypic information of the ASD patients included the ADOS total score and module information; and (3) more than 25 ASD patients from each site. Some important site-specific demographic information and details of the scanning parameters about the three sites are summarized in Table 3.

**Table 3:** Subject demographics and scanning parameters of each site.

Site	NYU	USM	UCLA_1	Total
**Subject demographics**				
Number of subjects	78	56	40	174
Gender (male/female)	67/11	56/0	42/7	157/17
Age (mean ± SD)	14.59 ± 6.98	22.27 ± 6.87	12.86 ± 2.35	16.74 ± 7.28
ADOS total score (mean ± SD)	11.28 ± 4.11	13.25 ± 3.35	11.38 ± 3.75	11.70 ± 3.90
Proxy calibrated severity score (mean ± SD)	6.33 ± 2.14	7.38 ± 1.72	6.6 ± 2.12	6.63 ± 2.06
**Scanning parameters**				
TR (msec)	2000	2000	3000	
TE (msec)	15	28	28	
Flip angle (deg)	90	90	90	
Voxel Size (mm)	3.75 × 3.75 × 4	3.43 × 3.43 × 3	3 × 3 × 4	
Slices	33	40	34	
Thickness (mm)	4	3	4	
Volumes	180	240	120	
Scan Time (min)	6	8	6	

Abbreviations: ASD, autism spectrum disorder; SD, standard deviation; ADOS, Autism Diagnostic Observation Schedule. The ADOS total score could be searched using phenotypic data provided by ABIDE. The Proxy calibrated severity score is calculated by matching the ADOS total score, the individual’s age, and its ADOS module information from the lookup table provided by Gotham *et al*.^51^.

### Image preprocessing

The fMRI data were preprocessed by using the Analysis of Functional Neuro Images (AFNI) software^50^. The following preprocessing steps were performed on the rs-fMRI data of each subject: (1) removing the first 10 volumes; (2) correcting for head motion in the time series; (3) removing the skull; (4) spatial smoothing using a Gaussian kernel with a full width at half maximum (FWHM) of 6 mm; (5) bandpass filtering between 0.005–0.1 Hz; (6) removing linear and quadratic trends; (7) regression of nuisance signals (cerebrospinal fluid, white matter, and global signals); (8) normalizing to the Montreal Neurological Institute (MNI) space with a resolution of 3 × 3 × 3 mm^3^; and (9) regressing out six head motion signals to decrease their effects.

### Evaluation of severity scores

To investigate the relationship between the RSFCs and ASD severity, the severity scores needed to be determined. The severity of the core autism features is usually assessed using the ADOS and the ADI-R phenotyping measures. Although higher ADOS and ADI-R scores indicate that individuals have more severe symptoms of ASD, these scores were not normalized for this purpose^51^. The problem with the ADI-R score as a measure of severity is that nonverbal children cannot be scored on approximately 25% of the total ADI-R items, affecting the scores in the communication domain. The ADOS is a semi-structured autism diagnostic assessment that has shown strong estimation validity, and the ADOS raw total score is a common measure of ASD severity^51,52^. However, the ADOS is generated for diagnostic purposes and was not devised to perform a comparison of data. Since different modules are used to estimate the scores according to the developmental and language levels of the individual, the scores of different modules cannot be directly compared. The assessed scores are also influenced by age. To compare the relative severity of ASD across modules and time, Gotham *et al*.^51^ provided calibrated severity scores by standardizing the ADOS scores in a large sample of data. Calibrated severity scores are distributed more evenly across developmental levels and are less affected by individual demographics, facilitating comparison of the severity of ASD across developmental groups and age ranges.

The phenotypic information of ABIDE includes calibrated severity scores (ADOS_GOTHAM_SEVERITY), but some subjects did not have these scores and did not have the necessary information to calculate them. Moradi *et al*.^12^ derived a proxy calibrated severity score from available ADOS measures and used it to study the relationship between cortical thickness and disorder severity. Specifically, due to the small difference between the total of the social and communication ADOS scores (ADOS_TOTAL) and the weighted sum of the ADOS item scores, Moradi *et al*.^12^ approximated the calibrated severity scores by replacing the weighted sum of the ADOS item scores with the total of the social and communication ADOS scores (ADOS_TOTAL). The proxy calibrated severity score was finally retrieved by matching the ADOS_TOTAL score, individual’s age, and its ADOS module information from the lookup table provided by Gotham *et al*.^51^. In the current study, we used the proxy calibrated severity scores as the observed scores for regression to explore the relationship between the RSFCs and disorder severity.

### Selection of RSFCs

The brain was parcellated into 116 ROIs using the AAL brain template^53^. The mean fMRI time series was calculated by averaging the signals of all voxels within each ROI. Then, we computed the Pearson correlation coefficients between the average time series of any pair of the 116 ROIs, gaining a symmetric matrix. To improve the normality of the correlation coefficients, we used Fisher’s r-to-z transformation to transform the correlation coefficient matrix into a z-score symmetric matrix. Finally, the 6670 z-scores extracted from the upper triangular part of the symmetric matrix were used as the RSFCs between brain ROIs.

Given that not all RSFCs provide valuable information for severity estimation and that some RSFCs may even degrade the regression estimation results, we adopted Pearson correlation analysis to select effective RSFCs to estimate the ASD severity. The Pearson correlation coefficient between each RSFC and the observed score across subjects in the training set was calculated, and the RSFCs whose p-value of the correlation coefficient fell below a predefined threshold (P = 0.0023) were retained for ASD severity estimation. The optimum value of P was determined experimentally. Specifically, the determination of the cutoff P threshold went through two stages. In the first stage, we used nested LOOCV on the training set (N-1 subjects, where N is the number of subjects) to select the hyperparameter P threshold from the range of 0.0005–0.05 with a step size of 0.0005, and the optimal P threshold was determined according to the minimum mean square error (MSE) between the estimated and observed scores. This process is very time-consuming, and the selected P threshold in each nested LOOCV is different, but statistically, almost 80% of the cutoff P thresholds fall in {0.0020, 0.0025, 0.0030} (see Supplementary Table S5 for details). In the second stage, taking into account the experience of the previous experiments and the main aim of this study—“investigate the whole-brain RSFCs that contribute most to ASD severity”—, we set the P threshold in the range of 0.002–0.003 with a step size of 0.0001 and then directly used LOOCV to estimate the severity score. The best correlation value between the estimated and observed severity was obtained at P = 0.0023 (R = 0.50) (see Supplementary Table S6 for details).

### ASD severity estimation

Based on the selected RSFCs, we applied linear SVR to estimate the severity scores. SVR can be used for regression problems and shares the same principles as the support vector machine (SVM) for classification. An SVM maps training data from the original low-dimensional space to higher-dimensional feature space by using a kernel function. An optimal separating hyperplane is determined to maximize the distance between the decision boundary and the nearest training samples. When the SVM’s response variable is a real number, the SVM becomes SVR. SVR has been successfully applied to disease severity and behavioural parameter estimation in brain imaging^12,13,54,55^.

In SVR, the ASD severity score is approximated by a regression model represented by a weight vector *w* and a bias *b*, as shown below.

1 $$y\approx f(x)={w}^{T}\varphi (x)+b$$

where *x* is the estimation factor, a vector consisting of a subset of RSFCs from a subject, and $\varphi (\cdot )$ is kernel function; *f*(*x*) is the response variable, i.e., the estimated severity score, and *y* is the known proxy calibrated severity score. The goal of regression is to learn the function *f*(*x*) that is as close as possible to *y*.

To solve the regression problem, the *ε*-SVR algorithm is proposed. The *ε*-SVR algorithm allows for a maximum deviation of *ε* between *f*(*x*) and *y*, i.e., a tube with radius *ε* is constructed to centre on *f*(*x*). If the data point falls within the tube, it is considered to be estimated correctly, and no loss is calculated when determining the regression line. However, in general, there are still some data points located outside the tube or on the boundary, and the loss needs to be calculated. In this case, the training error is allowed to exceed *ε* by introducing relaxation variables .. and ${\xi }_{i}^{\ast }$, and the constraint condition can be obtained. Given training samples $\{({x}_{1},{y}_{1}),\cdots ,({x}_{l},{y}_{l})\}$, where ${x}_{i}\in {R}^{n}$ is an RSFC feature vector and ${y}_{i}\in {R}^{1}$ is an observed score (proxy calibrated severity score), *ε*-SVR is designed to solve the following constrained optimization problem.

2 $$\begin{array}{c}{\rm{\min }}\,\frac{1}{2}{\Vert w\Vert }^{2}+C\mathop{\sum }\limits_{i=1}^{l}({\xi }_{i}+{\xi }_{i}^{\ast })\\ s.t.\{\begin{array}{c}({w}^{T}\varphi ({x}_{i})+b)-{y}_{i}\le \varepsilon +{\xi }_{i}\\ {y}_{i}-({w}^{T}\varphi ({x}_{i})+b)\le \varepsilon +{\xi }_{i}^{\ast }\\ {\xi }_{i},{\xi }_{i}^{\ast }\ge 0,\varepsilon \ge 0,i=1,2,\cdots ,l.\end{array}\end{array}$$

here, the regularization term $\frac{1}{2}{\Vert w\Vert }^{2}$ prevents overfitting, and the parameter *C* controls the trade-off between data fitting and regularization. In this study, we adopted a linear kernel function and set the penalty parameter to the default value, 1.

To assess the estimation performance, the estimated scores were correlated with the observed scores, and the Pearson correlation value (R) and the MAE were obtained to assess the regression accuracy. The LIBSVM software package was used to perform the linear kernel SVR for estimating the ASD severity scores^56^. We employed a LOOCV scheme to evaluate the regression performance. In particular, one subject is left out as a testing set for each cross-validation, and the remaining subjects are used as the training set. Each subject has an opportunity to be the testing set, which can produce a fair estimate of the regression accuracy. Note that all the above-described steps (especially the selection of RSFCs with Pearson correlation analysis) were performed only on the training samples.

## Supplementary information

Supplementary Information.

## Data Availability

The datasets for this study can be found in the Autism Brain Imaging Data Exchange I (ABIDE I) (http://fcon_1000.projects.nitrc.org/indi/abide/).
