# Supplementary material for: Alterations of functional connectivities associated with autism spectrum disorder symptom severity: a multi-site study using multivariate pattern analysis
Source: Sci Rep. 2020 Mar 9;10:4330. doi: 10.1038/s41598-020-60702-2 (PMC7062843; doi:10.1038/s41598-020-60702-2)
Supplement: Supplementary file 1 — Supplementary Information. [file 41598_2020_60702_MOESM1_ESM.pdf]

Supplementary Information

Alterations of functional connectivities associated with autism spectrum disorder symptom severity: a multi-site study using multivariate pattern analysis

Xingdan Liu[1] and Huifang Huang[1*]
[1]School of Computer and Information Technology, Beijing Jiaotong University, Beijing 100044, China
* Corresponding. hfhuang@bjtu.edu.cn; huifangbj@hotmail.com

Supplementary Tables:

ROI (1-116)	Coordinates			Network number	Name (BA)	ROI (1-116)	Coordinates			Network number	Name (BA)
1	-38	-5	50	**4**	PreCG.L	59	-23	-59	58	**1**	SPG.L
2	41	-8	52	**4**	PreCG.R	60	26	-59	62	**1**	SPG.R
3	-18	34	42	**4**	SFGdor.L	61	-42	-45	46	**3**	IPL.L
4	21	31	43	**4**	SFGdor.R	62	46	-46	49	**3**	IPL.R
5	-16	47	-13	**4**	ORBsup.L	63	-55	-33	30	**1**	SMG.L
6	18	48	-14	**4**	ORBsup.R	64	57	-31	34	**1**	SMG.R
7	-33	32	35	**3**	MFG.L	65	-44	-60	35	**3**	ANG.L
8	37	33	34	**3**	MFG.R	66	45	-59	38	**3**	ANG.R
9	-30	50	-9	**3**	ORBmid.L	67	-7	-56	48	**4**	PCUN.L
10	33	52	-10	**3**	ORBmid.R	68	9	-56	43	**4**	PCUN.R
11	-48	12	19	**3**	IFGoperc.L	69	-7	-25	70	**1**	PCL.L
12	50	14	21	**3**	IFGoperc.R	70	7	-31	68	**1**	PCL.R
13	-45	29	13	**3**	IFGtriang.L	71	-11	11	9	**5**	CAU.L
14	50	30	14	**3**	IFGtriang.R	72	14	12	9	**5**	CAU.R
15	-35	30	-12	**3**	ORBinf.L	73	-23	3	2	**5**	PUT.L
16	41	32	-11	**3**	ORBinf.R	74	27	4	2	**5**	PUT.R
17	-47	-8	13	**1**	ROL.L	75	-17	0	0	**5**	PAL.L
18	52	-6	14	**1**	ROL.R	76	21	0	0	**5**	PAL.R
19	-5	4	61	**3**	SMA.L	77	-10	-17	7	**5**	THA.L
20	8	0	61	**1**	SMA.R	78	13	-17	8	**5**	THA.R
21	-8	15	-11	**5**	OLF.L	79	-41	-18	9	**1**	HES.L
22	10	15	-11	**4**	OLF.R	80	45	-17	10	**1**	HES.R
23	-4	49	30	**4**	SFGmed.L	81	-53	-20	7	**1**	STG.L
24	9	50	30	**4**	SFGmed.R	82	58	-21	6	**1**	STG.R
25	-5	54	-7	**4**	ORBsupmed.L	83	-39	15	-20	**3**	TPOsup.L
26	8	51	-7	**4**	ORBsupmed.R	84	48	14	-16	**1**	TPOsup.R
27	-5	37	-18	**4**	REC.L	85	-55	-33	-2	**4**	MTG.L
28	8	35	-18	**4**	REC.R	86	57	-37	-1	**4**	MTG.R
29	-35	6	3	**1**	INS.L	87	-36	14	-34	**5**	TPOmid.L
30	39	6	2	**1**	INS.R	88	44	14	-32	**5**	TPOmid.R
31	-4	35	13	**4**	ACG.L	89	-49	-28	-23	**3**	ITG.L

32	8	37	15	**4**	ACG.R	90	53	-31	-22	**4**	ITG.R
33	-5	-14	41	**5**	DCG.L	91	-36	-66	-28	**6**	CRBLCrus1.L
34	8	-8	39	**5**	DCG.R	92	37	-67	-29	**6**	CRBLCrus1.R
35	-4	-42	24	**4**	PCG.L	93	-28	-73	-38	**6**	CRBLCrus2.L
36	7	-41	21	**4**	PCG.R	94	32	-69	-39	**6**	CRBLCrus2.R
37	-25	-20	-10	**5**	HIP.L	95	-8	-37	-18	**6**	CRBL3.L
38	29	-19	-10	**5**	HIP.R	96	12	-34	-19	**6**	CRBL3.R
39	-21	-15	-20	**5**	PHG.L	97	-15	-43	-16	**6**	CRBL45.L
40	25	-15	-20	**5**	PHG.R	98	17	-42	-18	**6**	CRBL45.R
41	-23	0	-17	**5**	AMYG.L	99	-23	-59	-22	**6**	CRBL6.L
42	27	0	-17	**5**	AMYG.R	100	24	-58	-23	**6**	CRBL6.R
43	-7	-78	6	**2**	CAL.L	101	-32	-59	-45	**6**	CRBL7b.L
44	15	-73	9	**2**	CAL.R	102	33	-63	-48	**6**	CRBL7b.R
45	-5	-80	27	**2**	CUN.L	103	-25	-54	-47	**6**	CRBL8.L
46	13	-79	28	**2**	CUN.R	104	25	-56	-49	**6**	CRBL8.R
47	-14	-67	-4	**2**	LING.L	105	-10	-48	-45	**6**	CRBL9.L
48	16	-66	-3	**2**	LING.R	106	9	-49	-46	**6**	CRBL9.R
49	-16	-84	28	**2**	SOG.L	107	-22	-33	-41	**6**	CRBL10.L
50	24	-80	30	**2**	SOG.R	108	25	-33	-41	**6**	CRBL10.R
51	-32	-80	16	**2**	MOG.L	109	0	-38	-20	**6**	Vermis12
52	37	-79	19	**2**	MOG.R	110	1	-39	-11	**6**	Vermis3
53	-36	-78	-7	**2**	IOG.L	111	1	-52	-6	**6**	Vermis45
54	38	-81	-7	**2**	IOG.R	112	1	-67	-15	**6**	Vermis6
55	-31	-40	-20	**2**	FFG.L	113	1	-71	-25	**6**	Vermis7
56	33	-39	-20	**2**	FFG.R	114	1	-64	-34	**6**	Vermis8
57	-42	-22	48	**1**	PoCG.L	115	0	-54	-34	**6**	Vermis9
58	41	-25	52	**1**	PoCG.R	116	0	-45	-31	**6**	Vermis10

Supplementary Table S1. The 116 ROIs in the AAL template are divided into 6 networks. The information of this table comes from the file ("Node_AAL116.node") provided by BrainNet Viewer software. "Coordinates" refer to the AAL coordinate. The "Network number" column is the serial number of the six networks (1-6): Network 1: SMN; Network 2: Visual; Network 3: EAN; Network 4: DMN; Network 5: SBC; Network 6: Cerebellum.

ROI 1	Name (BA)	ROI 2	Name (BA)	Times (174 loops)	Contribution	ROI 1	Name (BA)	ROI 2	Name (BA)	Times (174 loops)	Contribution
**1**	PreCG.L	**112**	Vermis_6	174	**1**	**66**	ANG.R	**80**	HES.R	9	0.05
**17**	ROL.L	**80**	HES.R	174	**1**	**33**	DCG.L	**85**	MTG.L	8	0.05
**17**	ROL.L	**82**	STG.R	174	**1**	**43**	CAL.L	**69**	PCL.L	6	0.03
**21**	OLF.L	**54**	IOG.R	174	**1**	**42**	AMYG.R	**107**	Cerebelum_10_L	5	0.03
**23**	SFGmed.L	**57**	PoCG.L	174	**1**	**81**	STG.L	**110**	Vermis_3	5	0.03
**24**	SFGmed.R	**57**	PoCG.L	174	**1**	**43**	CAL.L	**70**	PCL.R	4	0.02
**27**	REC.L	**41**	AMYG.L	174	**1**	**73**	PUT.L	**76**	PAL.R	4	0.02
**66**	ANG.R	**79**	HES.L	174	**1**	**17**	ROL.L	**30**	INS.R	3	0.02
**73**	PUT.L	**107**	Cerebelum_10_L	174	**1**	**18**	ROL.R	**65**	ANG.L	3	0.02
**74**	PUT.R	**107**	Cerebelum_10_L	174	**1**	**26**	ORBsupmed.R	**112**	Vermis_6	3	0.02
**76**	PAL.R	**107**	Cerebelum_10_L	174	**1**	**28**	REC.R	**41**	AMYG.L	3	0.02
**79**	HES.L	**80**	HES.R	174	**1**	**61**	IPL.L	**84**	TPOsup.R	3	0.02
**79**	HES.L	**82**	STG.R	174	**1**	**29**	INS.L	**80**	HES.R	2	0.01
**80**	HES.R	**81**	STG.L	174	**1**	**30**	INS.R	**65**	ANG.L	2	0.01
**81**	STG.L	**82**	STG.R	174	**1**	**35**	PCG.L	**67**	PCUN.L	2	0.01
**31**	ACG.L	**86**	MTG.R	173	**0.99**	**73**	PUT.L	**74**	PUT.R	2	0.01
**37**	HIP.L	**39**	PHG.L	173	**0.99**	**1**	PreCG.L	**24**	SFGmed.R	1	0.01
**47**	LING.L	**69**	PCL.L	173	**0.99**	**1**	PreCG.L	**57**	PoCG.L	1	0.01
**47**	LING.L	**70**	PCL.R	173	**0.99**	**5**	ORBsup.L	**90**	ITG.R	1	0.01
**7**	MFG.L	**24**	SFGmed.R	172	**0.99**	**12**	IFGoperc.R	**112**	Vermis_6	1	0.01
**1**	PreCG.L	**23**	SFGmed.L	171	**0.98**	**20**	SMA.R	**112**	Vermis_6	1	0.01
**7**	MFG.L	**85**	MTG.L	170	**0.98**	**24**	SFGmed.R	**79**	HES.L	1	0.01
**17**	ROL.L	**110**	Vermis_3	162	**0.93**	**27**	REC.L	**45**	CUN.L	1	0.01
**31**	ACG.L	**85**	MTG.L	152	**0.87**	**30**	INS.R	**80**	HES.R	1	0.01
**48**	LING.R	**69**	PCL.L	148	**0.85**	**40**	PHG.R	**86**	MTG.R	1	0.01
**7**	MFG.L	**86**	MTG.R	134	**0.77**	**65**	ANG.L	**80**	HES.R	1	0.01
**37**	HIP.L	**40**	PHG.R	111	**0.64**	**75**	PAL.L	**107**	Cerebelum_10_L	1	0.01
**25**	ORBsupmed.L	**112**	Vermis_6	32	0.18	**88**	TPOmid.R	**109**	Vermis_1_2	1	0.01
**38**	HIP.R	**86**	MTG.R	17	0.1	**95**	Cerebelum_3_L	**107**	Cerebelum_10_L	1	0.01
**51**	MOG.L	**69**	PCL.L	12	0.07	**104**	Cerebelum_8_R	**105**	Cerebelum_9_L	1	0.01
**30**	INS.R	**79**	HES.L	11	0.06	**104**	Cerebelum_8_R	**106**	Cerebelum_9_R	1	0.01

Supplementary Table S2. Details on all 62 RSFCs that were selected at least once in the LOOCV loops.

P threshold	Mean_R	Mean_MAE
0.0020	0.33	1.59
0.0021	0.34	1.59
0.0022	0.35	1.58
**0.0023**	**0.36**	**1.57**
0.0024	0.35	1.58
0.0025	0.34	1.59
0.0026	0.33	1.59
0.0027	0.33	1.59
0.0028	0.34	1.59
0.0029	0.34	1.59
0.0030	0.34	1.59

Supplementary Table S3. Mean results of 10-times 10-fold CV under different P thresholds. The experiment setting is the same as that for the LOOCV.

62 RSFCs selected in LOOCV

Number	RSFC	Contribution	Number	RSFC	Contribution
**1**	111	1	**28**	2571	0.18
**2**	1783	1	**29**	3637	0.1
**3**	1785	1	**30**	4543	0.07
**4**	2143	1	**31**	2978	0.06
**5**	2333	1	**32**	5409	0.05
**6**	2425	1	**33**	3236	0.05
**7**	2679	1	**34**	3995	0.03
**8**	5408	1	**35**	3960	0.03
**9**	5758	1	**36**	6069	0.03
**10**	5800	1	**37**	3996	0.02
**11**	5881	1	**38**	5727	0.02
**12**	5968	1	**39**	1733	0.02
**13**	5970	1	**40**	1866	0.02
**14**	6005	1	**41**	2661	0.02
**15**	6041	1	**42**	2767	0.02
**16**	3070	0.99	**43**	5153	0.02
**17**	3512	0.99	**44**	2893	0.01
**18**	4277	0.99	**45**	2964	0.01
**19**	4278	0.99	**46**	3381	0.01
**20**	692	0.99	**47**	5725	0.01
**21**	22	0.98	**48**	23	0.01
**22**	753	0.98	**49**	56	0.01
**23**	1813	0.93	**50**	539	0.01
**24**	3069	0.87	**51**	1310	0.01
**25**	4345	0.85	**52**	2106	0.01
**26**	754	0.77	**53**	2447	0.01
**27**	3513	0.64	**54**	2683	0.01
			**55**	2979	0.01
			**56**	3790	0.01
			**57**	5359	0.01
			**58**	5841	0.01
			**59**	6285	0.01
			**60**	6451	0.01
			**61**	6593	0.01
			**62**	6594	0.01

120 RSFCs selected in 10-fold CV

Number	RSFC	Contribution	Number	RSFC	Contribution
**1**	2425	1	**28**	3637	0.24
**2**	5800	1	**29**	3236	0.23
**3**	5968	1	**30**	4543	0.22
**4**	5970	0.99	**31**	3960	0.2
**5**	5881	0.98	**32**	2571	0.19
**6**	1785	0.97	**33**	2978	0.18
**7**	5758	0.97	**34**	3995	0.18
**8**	6041	0.96	**35**	5409	0.18
**9**	6005	0.92	**36**	1733	0.15
**10**	1783	0.88	**37**	2661	0.14
**11**	5408	0.84	**38**	1866	0.13
**12**	3512	0.74	**39**	2964	0.13
**13**	2333	0.73	**40**	3381	0.13
**14**	3070	0.73	**41**	5153	0.13
**15**	2143	0.72	**42**	6069	0.13
**16**	111	0.7	**43**	3996	0.12
**17**	4278	0.7	**44**	1310	0.11
**18**	692	0.63	**45**	2106	0.11
**19**	2679	0.63	**46**	2683	0.1
**20**	4277	0.61	**47**	3790	0.09
**21**	22	0.56	**48**	5359	0.09
**22**	753	0.52	**49**	5727	0.09
**23**	1813	0.48	**50**	6451	0.09
**24**	4345	0.42	**51**	2767	0.08
**25**	754	0.36	**...**	**...**	**...**
**26**	3069	0.36	**53**	5841	0.08
**27**	3513	0.36	**54**	2893	0.07
			**55**	5725	0.07
			**...**	**...**	**...**
			**57**	6285	0.07
			**58**	23	0.06
			**...**	**...**	**...**
			**60**	2447	0.06
			**61**	2979	0.06
			**...**	**...**	**...**
			**67**	56	0.04
			**...**	**...**	**...**
			**70**	6594	0.04
			**...**	**...**	**...**
			**93**	539	0.01
			**...**	**...**	**...**
			**117**	6593	0.01
			...	...	...

Supplementary Table S4. The 62 RSFCs selected in the LOOCV (left) and the 120 RSFCs selected in the 10-fold CV. The 10-fold CV was repeated ten times to estimate the severity of ASD. The RSFCs were sorted by their contribution. Specifically, all 62 RSFCs selected in the LOOCV were all selected in 10-fold CV (120 RSFCs), and the top 27 RSFCs in both the LOOCV and the 10-fold CV were the same, which demonstrates that the selected RSFCs from the LOOCV are still robust in the 10-fold CV.

Selected P threshold	Times (174 loops)	Frequency	Cumulative summation
0.0005	2	1.15%	1.15%
**0.0020**	**8**	**4.60%**	5.75%
**0.0025**	**97**	**55.75%**	61.49%
**0.0030**	**32**	**18.39%**	**79.89%**
0.0035	16	9.20%	89.08%
0.0040	11	6.32%	95.40%
0.0045	4	2.30%	97.70%
0.0050	3	1.72%	99.43%
0.0085	1	0.57%	100.00%

Supplementary Table S5. Determination of cutoff P threshold during the first stage in the nested LOOCV.

P threshold	R	MAE
0.0020	0.46	1.46
0.0021	0.47	1.44
0.0022	0.48	1.42
**0.0023**	**0.50**	**1.41**
0.0024	0.49	1.42
0.0025	0.49	1.43
0.0026	0.47	1.45
0.0027	0.46	1.46
0.0028	0.46	1.46
0.0029	0.47	1.44
0.0030	0.46	1.46

Supplementary Table S6. Results of the LOOCV under different P thresholds.

Supplementary Figure:

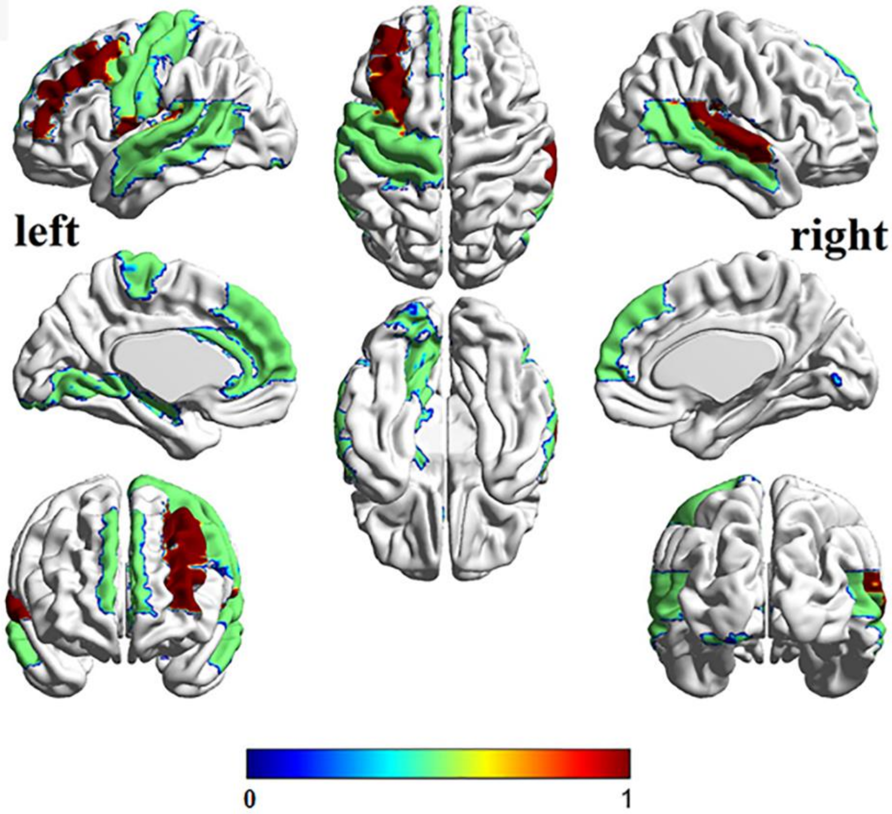

Supplementary Figure S1. Surface rendering map of all 59 ROIs associated with the 62 RSFCs selected by the LOOCV. The contribution of each ROI is evaluated by the contributions of all the RSFCs associated with it, and the colour ribbon below shows the scale for the normalized contributions of the ROIs.
